# The Effect of Shade and Thickness on the Depth of Cure of Bulk-Fill Composites with Different Viscosities

**DOI:** 10.30476/DENTJODS.2020.83927.1061

**Published:** 2020-12

**Authors:** Maryam Novin Rooz, Atefeh Yousefi Jordehi

**Affiliations:** 1 Dentist, Dept. of Operative Dentistry, School of Dentistry, Zanjan University of Medical Sciences, Zanjan, Iran; 2 Dept. of Operative Dentistry, School of Dentistry, Zanjan University of Medical Sciences, Zanjan, Iran

**Keywords:** Bulk-fill composites, Microhardness, Thickness, Depth of cure, Shade

## Abstract

**Statement of the Problem:**

In an attempt to enhance and simplify the restoration process, a new class of composite resins, called the bulk fill composite resins have been introduced. It is claimed that a depth of cure (DOC) of 4 mm can be achieved without affecting the properties of this material.

**Purpose:**

The purpose of this study was to investigate the effect of different shades, thicknesses, and viscosities on the DOC of bulk-fill composites.

**Materials and Method:**

In this experimental study, four bulk-fill composites [Filtek™ Bulk Fill Flowable (FBF), Filtek™ Bulk Fill posterior (FBP), Tetric® N-Flow Bulk Fill (TNF), Tetric® N-Ceram Bulk Fill (TNC)] and a conventional composite, Filtek™ Z250 XT Universal (FZ) were evaluated. The samples (n=5) were made using two different shades (light and dark), thicknesses (2 and 4mm), and viscosities (flowable and sculptable). Microhardness test was conducted on top and bottom surface using Vickers microhardness tester and DOC was calculated as the bottom/top ratio of yielded scores. Statistical analysis was done using a Mann Whitney test at *p*< 0.05.

**Results:**

DOC ranged between 52-95%. FBF composite exhibited the lowest overall hardness numbers. At 2-mm thickness, all the samples achieved an appropriate DOC. However, at 4mm thickness, only the light shades for FBF and TNF samples achieved a DOC very close to 0.8. At 4-mm thickness, the light shades for FBF, TNF and FZ samples exhibited significantly higher DOC compared to dark shades. For 4-mm-thick samples, the DOC of Filtek™ Bulk Fill (dark and light shades (and the DOC of Tetric® Bulk Fill (light shade (were different in flowable type from the sculptable type.

**Conclusion:**

The shade and the viscosity of bulk-fill composites influence their DOC at 4-mm depths. Moreover, 20 seconds of light curing appears insufficient for 4mm thickness of bulk-fill composite.

## Introduction

Currently, direct composite resins are the preferred materials for restoring small to medium cavities in posterior teeth on conditions in which the bonding and filling procedures can be properly performed [ 1 ]. Conventionally, to restore cavities with incremental technique, the composite resin is cured at a maximum thickness of 2mm. The main advantage of this technique is optimal cure throughout the material depth and decreased polymerization shrinkage [ 2 ]. On the other hand, the incremental technique is time-consuming, with higher risk of air bubbles being trapped between the layers and contamination of the operating field due to increased working time [ 3 ].

Recent developments in the technology of composite resin production have led to the introduction of bulk-fill composite resins, which can be cured in a thickness of 4-5 mm, resulting in a decrease in the duration of the restorative procedure [ 4 ]. Various studies have evaluated the physical properties of bulk-fill composites resins, including creep [ 5 ], modulus of elasticity [ 6 ], cuspal deflection [ 7 ], microleakage [ 8 ], and wear resistance [ 9 ].

As a material classification, the assessed mechanical properties put the bulk-fill composite resins between the nanohybrid and microhybrid composite resins and the flowable composite resins, signifying a parallel or even lower clinical performance of bulk-fill composite resins compared to nanohybrid and microhybrid composite resins [ 4 ]. Bulk-fill composite resins are also comparable with conventional composite resins considering water uptake and biocompatibility [ 10 ].

One of the most important factors in the failure of composite resin restorations is inadequate curing. Uncured composite resins might result in the failure of restoration because of increased chance of fracture, recurrence of caries or wear of the restoration. On the other hand, when the composite resin is not adequately cured, there is an increased risk of leakage of chemical materials from composite resin into the body tissues [ 11 ]. According to previous studies, the type of composite resin photoinitiator, filler type, matrix, color, translucency, light spectrum of the light-curing unit, and composite placement technique affect the depth of cure (DOC) of composite resins [ 12 ]. In addition, the thickness of the composite resin, irradiation time and the intensity of light influence the degree of conversion [ 13 ]. 

Typically, there are some methods for evaluating the adequate curing for a resin including degree of conversion using Fourier-transform infrared (FTIR) spectroscopy, and microhardness test. The majority of studies have indicated a good correlation between the degree of conversion and the microhardness test [ 14 - 16 ]. In the microhardness test, optimal DOC is defined as a depth with a hardness ratio of at least 0.8 of the hardness of composite resin surface [ 15 - 17 ]. Some researchers recommend the cure of bulk-fill composite resins at a thickness of 4 mm [ 18 - 19 ]; others believe that the methods used in reported studies have overestimated DOC of these composite resins and proclaim that the polymerization of bulk-fill composite resins at 4-mm depths is inadequate [ 11 , 20 ].

Bulk-fill composite resins are divided into two groups based on viscosity: bulk-fill composite resins with low viscosity (flowable) and bulk-fill composite resins with high viscosity (sculptable). 

The aim of this study is to evaluate the effect of viscosity, shade, and thickness on the DOC of bulk-fill composite resins. The null hypothesis states that DOC of the evaluated composite resins is not affected by viscosity, shade, and thickness.

## Materials and Method

**Figure 1:**
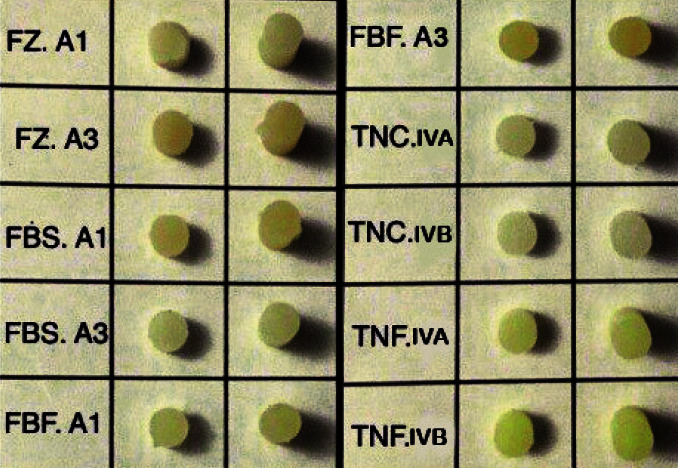
Resin composite samples

In this experimental study, four types of bulk-fill composite resins were evaluated: FiltekTM Bulk Fill Flowable (3M ESPE, St Paul, MN, USA) (FBF), Filtek^TM^ Bulk Fill Posterior (3M ESPE, St Paul, MN, USA) (FBP), Tetric® N-Ceram Bulk Fill (Ivoclar Vivadent, Schaan, Liechtenstein) (TNC), and Tetric® N-Flow Bulk Fill (Ivoclar Vivadent, Schaan, Liechtenstein) (TNF). Moreover, a conventional composite resin, Filtek^TM^ Z250XT (Nano Hybrid Universal, 3M ESPE, St Paul, MN, USA) (FZ) was also evaluated (Figure 1). Composition and brands of materials are listed in Table 1.

**Table 1:** Resin composites used in this study

Composite	Abbreviation	Manufacturer	Bulk Fill kind	Shade	Resin matrix	Filler	Filler Content (wt%)	Recommendation
Tetric N-Ceram Bulk Fill	TNC	Ivoclar Vivadent, Schaan, Liechtenstein	Bulk Fill posterior restorative	IVA, IVB	BisGMA, UDMA	Barium glass, prepolymer, YbF3, mixed oxide	75-77%	4mm
								20s
								≥500mW/cm^2^
								10s
								≥1,000mW/cm^2^
Tetric N-Flow Bulk Fill	TNF	Ivoclar Vivadent, Schaan, Liechtenstein	Bulk Fill Flowable base	IVA, IVB	Monomethacrylates, Dimethacrylates	Barium glass, YbF3, copolymers	68.2%	4mm
								20s
								≥500mW/cm^2^
								10s
								≥1,000mW/cm^2^
Filtek Bulk Fill Flowable	FBF	3M ESPE, St Paul, MN, USA	Bulk Fill Flowable base	A1, A3	BisGMA, UDMA, BisEMA, Procrylat resins	YbF3	64.5%	4mm
								40s
								550-1,000 mW/cm^2^
								20s
								1,000-2,000 mW/cm^2^
Filtek Bulk Fill Posterior	FBP	3M ESPE, St Paul, MN, USA	Bulk Fill posterior restorative	A1, A3	ERGP-DMA, Diurethane-DMA, DDDMA	Silica, zirconia, aggregated zirconia/silica cluster, YbF3	76.5%	4mm
								40s
								550-1,000 mW/cm^2^
								20s
								1,000-2,000 mW/cm^2^
Filtek Z-250 Universal	FZ	3M ESPE, St Paul, MN, USA	Conventional sculptable	A1, A3	BIS-GMA, UDMA and BIS-EMA, TEGDMA	Zirconia/silica	60%	2mm
								20s
								≥400mW/cm^2^

Abbreviations: BIS-GMA, bisphenol A dimethacrylate; BIS-EMA, bisphenol A Polyethylene Glycol Diether Dimethacrylate; UDMA, urethane dimethacrylate;

TEGDMA,triethylene glycol dimethacrylate; DDDMA,1,12-dodecane-DMA; YbF3, Ytterbium Trifluoride

A1 shade was considered as a light shade (L) for FZ, FBP and FBF composite resins and IVA shade was considered as a light shade (L) for TNC and TNF composite resins. In addition, the A3 shade was considered as a dark shade for FBF, FBP and FZ composite resins and IVB shade was considered as a dark shade for TNC and TNF composite resins (D).

The samples were assigned to 20 groups and five samples were prepared for each group [ 21 ]. Steel molds, measuring 4 mm in diameter and 2 or 4 mm in thickness were used to prepare the samples [ 22 ]. After placing the mold on a glass slab and celluloid matrix strip, the composite resin was packed within it; then a glass slab and a celluloid matrix strip were placed on the upper surface of the composite resin and the excess material was removed by exerting pressure on the glass slab.

The samples were light-cured for 20s using a Polywave LED light-curing unit (Bluephase N, Ivoclar Vivadent, Schaan, Liechtenstein) in "high" mode. Radiation intensity was measured with a radiometer (Ivoclar Vivadent, Schaan, Liechtenstein) before each curing procedure. The intensity of the radiation was 1200±40 mW/cm^2^ during each curing procedure.

All the samples were incubated (Peco, Iran, Model: PI-455G) at 37°C in a dry environment within a lightproof container for 24 hours [ 19 ]. Then, the microhardness of the samples was measured by Vickers hardness machine (ZHVµ model, Zwick/Roel, United Kingdom). To measure the microhardness, first the samples were placed on the jig of the device and their surface was evaluated at ×40 magnification so that the location of the force on the surface was free of bubbles and other defects. Then a 300-gr load was applied to the sample for 10 seconds by a diamond pyramid-shaped indenter [ 23 ]. The loads were applied close to the center of the samples at a distance of 0.2 mm of each other. Then with adjusting the electronic microscope index on the surface of sample, the diameter of the square indentation area was determined by tester. Finally, the surface and bottom Vickers microhardness of specimens’ calculations were made using computer processor of tester device using this formula: VHN = (1.8544P) / D2.

 In this equation, VHN represents Vickers hardness of material (Kg/mm2), P is the predetermined load applied on the sample (Kg) and D is the average diagonal distance (mm) of the square resulting from indentation of the pyramid tip of Vickers hardness tester [ 15 ]. 

To obtain the hardness value of each surface, three measurements were made on each surface and their mean was determined and recorded as the final hardness score for each surface. For each sample, two hardness scores were obtained, which belonged to the top and the bottom of the samples. Then, by calculating bottom/top ratio, DOC of the samples was determined [ 11 , 19 ] and the results were analyzed.

Kolmogorov-Smirnov test was used to evaluate the normality of data. Since data were not normal, Mann-Whitney test was used to compare each variable individually. SPSS 24 was used for statistical analysis at a significance level of P<0.05.

## Results

Table 2 presents the mean hardness of the top and the bottom for each sample in terms of shade, viscosity and thickness.

**Table 2:** Bottom and top means and standard deviations (SD) of Vickers hardness scores of different resin-based composite

		4mm,dark	4mm,light	2mm,dark	2mm,light
FZ	Top	87.73(4.81)	87.86(2.03)	84.13(1.32)	90.59(2.51)
	Bottom	45.99(5.74)	58.86(5.38)	80.39(3.51)	81.99(5.22)
FBP	Top	63.86(1.32)	59.99(2.23)	63.80(1.32)	62.59(1.53)
	Bottom	46.86(2.54)	42.26(2.08)	60.66(2.41)	59.66(2.53)
FBF	Top	26.74(0.73)	29.13(1.14)	29.99(0.62)	29.33(1.43)
	Bottom	16.99(0.97)	23.19(1.30)	27.73(0.54)	28.06(0.89)
TNC	Top	55.53(2.00)	55.79(1.07)	56.73(0.59)	51.16(1.86)
	Bottom	36.32(3.83)	38.06(1.70)	51.73(0.75)	51.06(0.49)
TNF	Top	37.66(1.35)	34.39(1.69)	36.59(2.68)	34.73(1.94)
	Bottom	27.26(2.17)	27.46(1.42)	34.46(2.40)	32.92(1.90)

FZ:Filtek Z250 A1,A3; FBP: Filtek Bulk Fill Posterior A1,A3; FBF: Filtek Bulk Fill Flowable A1,A3; TNC: Tetric N-Ceram Bulk Fill IVA,IVB; TNF:Tetric N-Flow Bulk Fill IVA,IVB

**Figure 2:**
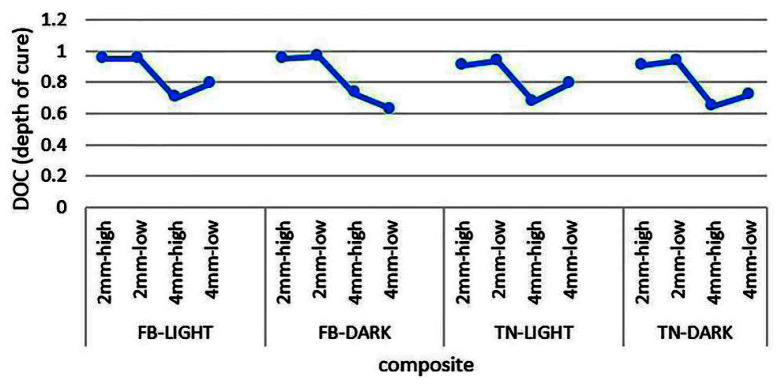
Graph presenting the DOC B/T ratios of groups comparing thickness (2 and 4mm) and shades (light and dark)

**Figure 3:**
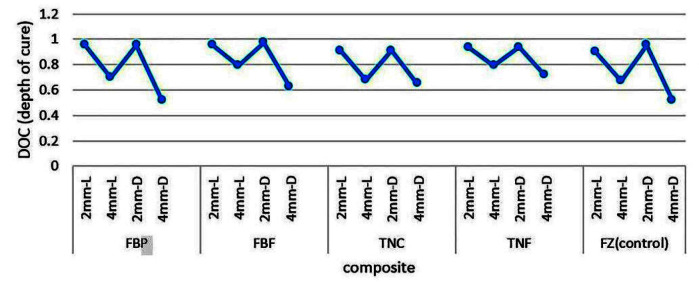
Graph presenting the DOC B/T ratios of dark and light shades comparing viscosity (high and low)

Among the composite resins evaluated, FBF composite resin exhibited the lowest hardness number at top and bottom; FZ composite resin exhibited the highest overall hardness values compared to the other materials. In addition, in all groups, the top hardness was higher than the bottom. According to statistical analyses, 2-mm-thick samples for all groups had significantly higher DOC compared 4-mm-thick samples (Figure 2). At 2-mm thickness, all the samples achieved an appropriate DOC (DOC>0.8). However, at 4-mm thickness, only FBF and TNF composite resins (light shades) achieved a DOC very close to 0.8. At 2-mm-thick samples, different shades had no effects on the DOC of various composite resins; however, when 4-mm-thick samples were evaluated, only the light shades for FBF and TNF samples achieved a DOC very close to 0.8 (Figure 2). Comparison of different viscosities of composite resins at 2-mm thickness showed no significant difference in DOC of groups in terms of the type of viscosity (Figure 3). For 4-mm-thick samples, DOC of FB (dark and light shades (and DOC of TN (light shade (were different in the flowable type from the sculptable type.

## Discussion

Bulk-fill composite resins, which can be cured at a thickness of 4-5 mm, were introduced to reduce the duration of restorative procedures [ 4 ]. Bulk-fill composite resins are cured in thicknesses greater than 2 mm due to some mechanisms. These include (1) booster photo initiators derived from benzoyl germanium with higher light-curing activity [ 24 ], (2) polymerization modulators, such as urethane-based dimethacrylate monomers with high molecular weight, which reduce the stresses of polymerization [ 25 ], (3) increased flowability for better adaptation [ 3 ], and (4) increased translucency through the use of mixed oxide fillers with refractive indexes equal to the resin matrix and use of glass fibers that increase the penetration of light into the composite resin [ 26 ].

Some studies evaluated the impact of different shades on the DOC and stated that darker shades presented lower microhardness than light shades. It has been verified that different composite resin compositions, filler size, weight, volume, and filler-to-matrix ratio have a considerable effect on the composite resins’ DC and microhardness [ 27 ]. Therefore, we concurrently evaluated the effect of viscosity, shade, and thickness on the DOC of bulk-fill composite resins. 

Consistent with the results of previous studies [ 19 - 20 ], hardness of the top was higher than that of the bottom. FZ composite resin exhibited the highest surface hardness, followed by FBP composite resin. In line with the results of previous studies [ 20 , 28 ], in the current study, the surface hardness in the flowable type was significantly lower than that in the sculptable type in each composite resin, which could be due to the lower filler content of flowable composite resins. Studies have shown that the filler content of composite resins could affect their hardness and physico-mechanical properties [ 29 ]. The results of this study showed lower DOC in all the composite resins in 4-mm- thick samples compared to 2-mm-thick samples (Table 2), consistent with the results of previous studies [ 19 - 20 ]. A likely rationale could be the absence of light penetration through the composite at increasing depths since a high percentage of the wavelengths are absorbed in approximate to the top surface of the composite, subsequently it cannot excite co-initiators at larger depths [ 30 ]. 

Generally, manufacturers use methods such as in creasing translucency, increasing the amount of photo initiators, and use of additional photo initiators to increase DOC of composite resins [ 31 ]. In this context, these composite resins have less light attenuation and more light transition rates compared to conventional composite resins [ 20 ].

In agreement with the results of previous studies [ 11 ], in our study, all the 2-mm-thick composite resin samples reached an adequate DOC (0.8) with a maximum curing time of 20 seconds. However, none of the 4-mm-thick samples in any group reached a DOC of 0.8, except for the light shades of flowable composite resins (FBF A1 and TF IVA), in which the DOC was very close to 0.8 (0.79). This finding is contrary to the claims made by the manufacturers about the DOC of composite resins at 4-mm thickness. It seems that the scraping ISO 4049 method has overestimated the DOC [ 22 ] and also it is hard to standardize it [ 32 ]; the current study employed the Vickers hardness test to examine the DOC.

 Several studies have delineated DOC considering hardness measurements performed on the top and bottom surface of a light-cured resin composite specimen and reported a ratio of 0.80 to be regarded as a crucial minimum acceptable threshold value [ 33 - 35 ]. However, some studies have reported a DOC of >0.8 for 4-mm-thick bulk-fill composite resin samples, which is acceptable [ 28 ]. Other studies, consistent with the present study, have reported DOC of <0.8 in 4-mm-thick samples of the bulk-fill composite resins [ 19 - 20 , 22 ]. The differences in the results of these studies might be attributed to the differences in sample preparation conditions, storage of the samples, the method used to determine the DOC, composite resin type, mold type and diameter, the use of a lubricant, the intensity of the light-curing unit, the storage conditions of the samples, the method of testing, and the amount of load used in different studies [ 11 ]. 

The impact of mold size has been studied for opaque cylindrical molds and the results showed that DOC would decrease if the mold size diameter were decreased [ 36 ]. Black molds presented shorter DOC than a stainless steel mold once a light shade of composite was cured [ 37 ].

When a mold is used, a prominent effect by the walls would cause decreased hardness as the mold wall is approached and the severity of this effect was associated with the color of the mold. It can be stated that this incident is because of absorption/reflection properties of light by the walls, with the white molds presenting the least effect [ 38 ].

The mold used in our research was made out of metal. This would block the transmission of the all lights outside of the central 4-mm of the light guide tip. Nevertheless, a metal mold is defined in the ISO standard 4049 and has been suggested by different studies for an accurate measurement of DOC [ 36 , 38 - 39 ]. This metal mold brought the experimental conditions more similar to clinical situations where a metallic matrix band is placed around the boxes in Class 2 preparations [ 11 ].

The results of this study indicated no significant differences in the curing depths between the dark and light shades except between two flowable bulk-fill composite resins with 4-mm thickness. However, Rodriguez *et al*. [ 19 ] concluded that when Tetric EvoCeram Bulk Fill and SonicFill™ composite resin samples with 4-mm thickness were light-cured for 20 seconds, there was a significant difference in curing depth between dark and light shades and DOC in light shades was greater than that in dark shades [ 40 ]. 

The size, radioactivity, translucency, and pigments in the filler particles affect light transmission of the material [ 41 ]. Pigments in dark shades limit the light transmission and reduce the degree of polymerization [ 30 ]. It seems that due to lower filler content and higher translucency of flowable bulk-fill composite resins compared to sculptable type, presence of more pigments in the dark shade resulted in a decrease in curing depth when the thickness of composite has been increased resins to 4 mm [ 42 ].

The results of this study indicated no significant difference in the DOC between flowable and sculptable composite resins at 2-mm thickness; however, at 4-mm thickness, in flowable type the DOC was significantly different from that in sculptable type in all the groups, except the one in IVB dark shade of TNC bulk-fill composite resin. Consistent with the results of this study, some researchers have reported greater DOC in flowable bulk-fill composite resins compared to sculptable composite resins [ 3 ]. This variation in the DOC of bulk-fill composite resins with different viscosities might be related to the difference in their filler content. By increasing filler-to-matrix ratio, the degree of conversion decreases since high filler content prevents the development of polymer chains [ 43 ]. In addition, as the amount of filler increases, the amount of light scattering increases and the translucency for the blue color decreases [ 26 ].

Investigating by scanning electron microscope, bulk-fill flowable showed large filler size with dominant polygonally shaped characteristics compared to conventional flowable resin composites. The filler load was slightly increased, however, because of the bigger size of the filler particle, the filler-matrix interface was supposed to be decreased. Therefore, it permits more curing light to transmit through the composite and improve the DOC [ 44 ].

In this study only four types of bulk-fill composite resins were studied; therefore, it is suggested that other bulk-fill composite resins should be studied and the effects of other variables, including the intensity of radiation, type of light-curing unit and its distance from the surface of composite resin, on their DOC could be investigated in future studies.

## Conclusion

The shade and the viscosity would influence the curing depth of bulk-fill composites at 4-mm depths. None of the composite resins investigated in this study reached a curing depth of >0.8 mm at 4-mm thickness. The samples of flowable composite resins in light shade exhibited a curing depth very close to 0.8. Under the limitations of this study, 20 seconds of light curing appears insufficient for curing the 4mm-thick bulk-fill composite. 
